# *n*-Tridecyl­amine chloride monohydrate

**DOI:** 10.1107/S1600536811006246

**Published:** 2011-02-26

**Authors:** Lijun Zhang, Youying Di, Wenyan Dan

**Affiliations:** a College of Chemistry and Chemical Engineering, Liaocheng University, Shandong 252059, People’s Republic of China

## Abstract

In the title compound, C_13_H_30_N^+^·Cl^−^·H_2_O, the C_13_H_27_ alkyl chain is in an all-*trans* conformation. In the crystal, inter­molecular N—H⋯Cl, N—H⋯O and O—H⋯Cl hydrogen bonds connect the components into layers parallel to (010), with the alkyl chains oriented approximately perpendicular to these layers.

## Related literature

For applications of long-chain *n*-alkyl­ammonium halides, see: Aratono *et al.* (1998 ▶); Tornblom *et al.* (2000 ▶); Ringsdorf *et al.* (1988 ▶). For details of phase transitions in *n-*alkyl­ammonium chlorides, see: Terreros *et al.* (2000 ▶). For related structures, see: Rademeyer *et al.* (2009 ▶); Lundén (1974 ▶); Clark & Hudgens (1950 ▶).
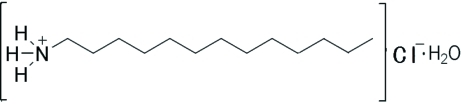


## Experimental

### 

#### Crystal data

C_13_H_30_N^+^·Cl^−^·H_2_O
*M* *_r_* = 253.85
Monoclinic, 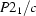

*a* = 4.7420 (5) Å
*b* = 45.250 (3) Å
*c* = 7.8191 (9) Å
β = 106.332 (2)°
*V* = 1610.1 (3) Å^3^
*Z* = 4
Mo *K*α radiation
μ = 0.22 mm^−1^
*T* = 298 K
0.34 × 0.33 × 0.03 mm

#### Data collection

Siemens SMART CCD area-detector diffractometer
Absorption correction: multi-scan (*SADABS*; Sheldrick, 1996 ▶) *T*_min_ = 0.928, *T*_max_ = 0.993
8230 measured reflections
2845 independent reflections
1379 reflections with *I* > 2σ(*I*)
*R*_int_ = 0.077

#### Refinement

*R*[*F* ^2^ > 2σ(*F* ^2^)] = 0.067
*wR*(*F* ^2^) = 0.124
*S* = 1.03
2845 reflections
147 parameters
H-atom parameters constrained
Δρ_max_ = 0.25 e Å^−3^
Δρ_min_ = −0.18 e Å^−3^

### 

Data collection: *SMART* (Siemens, 1996 ▶); cell refinement: *SAINT* (Siemens, 1996 ▶); data reduction: *SAINT*; program(s) used to solve structure: *SHELXS97* (Sheldrick, 2008 ▶); program(s) used to refine structure: *SHELXL97* (Sheldrick, 2008 ▶); molecular graphics: *SHELXTL* (Sheldrick, 2008 ▶); software used to prepare material for publication: *SHELXTL*.

## Supplementary Material

Crystal structure: contains datablocks I, global. DOI: 10.1107/S1600536811006246/lh5197sup1.cif

Structure factors: contains datablocks I. DOI: 10.1107/S1600536811006246/lh5197Isup2.hkl

Additional supplementary materials:  crystallographic information; 3D view; checkCIF report

## Figures and Tables

**Table 1:** Hydrogen-bond geometry (Å, °)

*D*—H⋯*A*	*D*—H	H⋯*A*	*D*⋯*A*	*D*—H⋯*A*
N1—H1*A*⋯Cl1	0.89	2.44	3.303 (3)	162
N1—H1*B*⋯Cl1^i^	0.89	2.36	3.236 (2)	170
N1—H1*C*⋯O1^ii^	0.89	2.05	2.901 (4)	159
O1—H1*H*⋯Cl1	0.85	2.45	3.290 (3)	170
O1—H1*I*⋯Cl1^iii^	0.85	2.39	3.228 (2)	170

Symmetry codes: (i) 
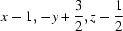
; (ii) 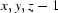; (iii) 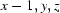.

**Fig. 1.**
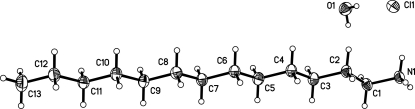
The molecular structure of the title compound with the atom numbering scheme. Displacement ellipsoids are drawn at the 30% probability level. H atoms are presented as a small spheres of arbitrary radius.

**Fig. 2.**
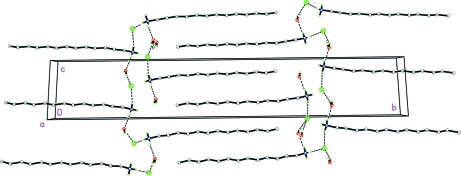
Part of the crystal structure with hydrogen bonds shown as dashed lines.

## supplementary crystallographic information

### Comment

Long-chain *n*-alkylammonium halides are widely used as surfactants (Aratono *et al.*, 1998; Tornblom *et al.*, 2000) and as models for biological membranes (Ringsdorf *et al.*, 1988). It has been shown that phase transitions occur in *n*-alkylammonium chlorides (Terreros *et al.*, 2000). As a part of our studies on novel potential phase transition materials with thermochemical properties, we report herein the crystal structure of the title compound (Fig. 1).

Atoms C2–C13 are essentially co-planar with a maximum deviation of 0.048 (3)Å for atom C2. The alkyl chain in related compounds is typically in the extended conformation e.g. in the isostructural *n*-tridecylamine bromide monohydrate compound (Rademeyer *et al.*, 2009), *n*–dodecylammonium bromide (Lundén, 1974) and *n*–tridecylamine chloride (Clark & Hudgens, 1950). Although the methylene chain has the extended all–*trans* conformation, it is slightly bent in the vicinity of the ammonium group possibly to accommodate the hydrogen–bonding interactions. Only the C1–C2–C3–C4 torsion angle deviates significantly from 180 °, with a value of 169.84 (3)°. The crystal packing (Fig. 2) is stabilized by intermolecular N—H···Cl, N—H···O and O—H···Cl hydrogen bonds (Table 1 and Fig.2).

### Experimental

*n*–Tridecylamine chloride monohydrate was prepared by the addition of hydrochloric acid to an ethanolic solution of *n*–tridecylamine. The mixture was heated and stirred under reflux for 6 h. Single crystals suitable for *X*–ray diffraction were prepared by evaporation of the resulting solution at room temperature. Analysis, calculated for C_13_H_32_ClNO (Mr =253.85): C 61.51, H 12.71, N 5.52, Cl 13.96%; found: C 61.50, H 12.72, N 5.51, Cl 13.95%.

### Refinement

All H atoms were placed in geometrically idealized positions and constrained to ride on their parent atoms, with methylene C—H = 0.97 Å, methyl C—H = 0.96 Å, N—H = 0.89 Å, O-H = 0.85 Å and refined as riding on their parent atoms. The*U*_iso_(H) values were set at 1.2*U*_eq_(C_methylene_, O) at 1.5*U*_eq_(C_methyl_,N).

### Crystal data

**Table:**

C_13_H_30_N^+^·Cl^−^·H_2_O	*F*(000) = 568
*M**_r_* = 253.85	*D*_x_ = 1.047 Mg m^−^^3^
Monoclinic, *P*2_1_/*c*	Mo *K*α radiation, λ = 0.71073 Å
Hall symbol: -P 2ybc	Cell parameters from 817 reflections
*a* = 4.7420 (5) Å	θ = 2.7–20.8°
*b* = 45.250 (3) Å	µ = 0.22 mm^−^^1^
*c* = 7.8191 (9) Å	*T* = 298 K
β = 106.332 (2)°	Acicular, colourless
*V* = 1610.1 (3) Å^3^	0.34 × 0.33 × 0.03 mm
*Z* = 4	

### Data collection

**Table:**

Siemens SMART CCD area-detector diffractometer	2845 independent reflections
Radiation source: fine-focus sealed tube	1379 reflections with *I* > 2σ(*I*)
graphite	*R*_int_ = 0.077
Detector resolution: 10 pixels mm^-1^	θ_max_ = 25.0°, θ_min_ = 2.7°
φ and ω scans	*h* = −5→5
Absorption correction: multi-scan (*SADABS*; Sheldrick, 1996)	*k* = −53→46
*T*_min_ = 0.928, *T*_max_ = 0.993	*l* = −7→9
8230 measured reflections	

### Refinement

**Table:**

Refinement on *F*^2^	Primary atom site location: structure-invariant direct methods
Least-squares matrix: full	Secondary atom site location: difference Fourier map
*R*[*F*^2^ > 2σ(*F*^2^)] = 0.067	Hydrogen site location: inferred from neighbouring sites
*wR*(*F*^2^) = 0.124	H-atom parameters constrained
*S* = 1.03	*w* = 1/[σ^2^(*F*_o_^2^) + (0.0256*P*)^2^] where *P* = (*F*_o_^2^ + 2*F*_c_^2^)/3
2845 reflections	(Δ/σ)_max_ = 0.001
147 parameters	Δρ_max_ = 0.25 e Å^−^^3^
0 restraints	Δρ_min_ = −0.18 e Å^−^^3^

### Special details

**Table:**

Geometry. All e.s.d.'s (except the e.s.d. in the dihedral angle between two l.s. planes) are estimated using the full covariance matrix. The cell e.s.d.'s are taken into account individually in the estimation of e.s.d.'s in distances, angles and torsion angles; correlations between e.s.d.'s in cell parameters are only used when they are defined by crystal symmetry. An approximate (isotropic) treatment of cell e.s.d.'s is used for estimating e.s.d.'s involving l.s. planes.
Refinement. Refinement of *F*^2^ against ALL reflections. The weighted *R*-factor *wR* and goodness of fit *S* are based on *F*^2^, conventional *R*-factors *R* are based on *F*, with *F* set to zero for negative *F*^2^. The threshold expression of *F*^2^ > σ(*F*^2^) is used only for calculating *R*-factors(gt) *etc*. and is not relevant to the choice of reflections for refinement. *R*-factors based on *F*^2^ are statistically about twice as large as those based on *F*, and *R*- factors based on ALL data will be even larger.

### Fractional atomic coordinates and isotropic or equivalent isotropic displacement parameters (Å^2^)

**Table:**

	*x*	*y*	*z*	*U*_iso_*/*U*_eq_	
Cl1	0.77004 (19)	0.727389 (19)	0.54882 (12)	0.0578 (3)	
N1	0.2908 (5)	0.72396 (5)	0.1504 (4)	0.0486 (8)	
H1A	0.4378	0.7281	0.2466	0.073*	
H1B	0.1648	0.7390	0.1270	0.073*	
H1C	0.3618	0.7210	0.0578	0.073*	
O1	0.3848 (5)	0.70538 (5)	0.8156 (3)	0.0690 (8)	
H1H	0.5034	0.7102	0.7566	0.083*	
H1I	0.2125	0.7103	0.7554	0.083*	
C1	0.1369 (7)	0.69683 (6)	0.1834 (4)	0.0462 (9)	
H1D	−0.0396	0.6941	0.0856	0.055*	
H1E	0.0779	0.6994	0.2915	0.055*	
C2	0.3263 (7)	0.66944 (6)	0.2017 (4)	0.0445 (9)	
H2A	0.5028	0.6722	0.2995	0.053*	
H2B	0.3852	0.6668	0.0935	0.053*	
C3	0.1677 (7)	0.64186 (6)	0.2355 (4)	0.0467 (9)	
H3A	−0.0251	0.6412	0.1498	0.056*	
H3B	0.1397	0.6431	0.3534	0.056*	
C4	0.3305 (7)	0.61334 (6)	0.2221 (4)	0.0444 (9)	
H4A	0.5222	0.6140	0.3090	0.053*	
H4B	0.3616	0.6123	0.1049	0.053*	
C5	0.1730 (7)	0.58533 (6)	0.2526 (4)	0.0471 (9)	
H5A	0.1495	0.5860	0.3718	0.056*	
H5B	−0.0219	0.5851	0.1691	0.056*	
C6	0.3292 (7)	0.55675 (6)	0.2317 (4)	0.0440 (9)	
H6A	0.3543	0.5562	0.1128	0.053*	
H6B	0.5234	0.5569	0.3159	0.053*	
C7	0.1722 (7)	0.52882 (6)	0.2603 (4)	0.0450 (9)	
H7A	−0.0231	0.5288	0.1773	0.054*	
H7B	0.1496	0.5292	0.3798	0.054*	
C8	0.3260 (6)	0.50019 (6)	0.2369 (4)	0.0440 (9)	
H8A	0.3473	0.4997	0.1171	0.053*	
H8B	0.5218	0.5003	0.3193	0.053*	
C9	0.1705 (7)	0.47222 (6)	0.2666 (4)	0.0448 (9)	
H9A	−0.0251	0.4721	0.1839	0.054*	
H9B	0.1485	0.4727	0.3862	0.054*	
C10	0.3245 (7)	0.44364 (6)	0.2439 (4)	0.0438 (9)	
H10A	0.3458	0.4431	0.1242	0.053*	
H10B	0.5204	0.4438	0.3263	0.053*	
C11	0.1702 (7)	0.41561 (6)	0.2745 (4)	0.0448 (9)	
H11A	0.1479	0.4162	0.3940	0.054*	
H11B	−0.0253	0.4154	0.1917	0.054*	
C12	0.3236 (7)	0.38729 (6)	0.2530 (5)	0.0522 (10)	
H12A	0.5194	0.3876	0.3355	0.063*	
H12B	0.3452	0.3867	0.1333	0.063*	
C13	0.1688 (8)	0.35920 (7)	0.2844 (5)	0.0703 (12)	
H13A	0.1532	0.3591	0.4042	0.105*	
H13B	0.2798	0.3423	0.2668	0.105*	
H13C	−0.0242	0.3584	0.2021	0.105*	

### Atomic displacement parameters (Å^2^)

**Table:**

	*U*^11^	*U*^22^	*U*^33^	*U*^12^	*U*^13^	*U*^23^
Cl1	0.0520 (5)	0.0590 (6)	0.0612 (6)	−0.0052 (5)	0.0137 (4)	−0.0036 (5)
N1	0.0485 (16)	0.0358 (17)	0.061 (2)	0.0041 (15)	0.0145 (14)	0.0002 (14)
O1	0.0659 (17)	0.0752 (17)	0.0683 (19)	0.0036 (15)	0.0230 (13)	0.0100 (14)
C1	0.044 (2)	0.034 (2)	0.062 (3)	0.0005 (18)	0.0178 (17)	0.0039 (16)
C2	0.046 (2)	0.035 (2)	0.053 (2)	−0.0022 (18)	0.0152 (17)	−0.0021 (16)
C3	0.052 (2)	0.039 (2)	0.053 (2)	−0.0043 (19)	0.0209 (18)	0.0011 (17)
C4	0.050 (2)	0.036 (2)	0.050 (2)	−0.0033 (18)	0.0191 (18)	0.0020 (16)
C5	0.053 (2)	0.041 (2)	0.049 (2)	−0.005 (2)	0.0189 (18)	0.0004 (17)
C6	0.048 (2)	0.039 (2)	0.048 (2)	−0.0014 (19)	0.0177 (17)	0.0012 (16)
C7	0.048 (2)	0.039 (2)	0.050 (2)	−0.0024 (19)	0.0178 (17)	0.0029 (17)
C8	0.047 (2)	0.040 (2)	0.047 (2)	−0.003 (2)	0.0170 (17)	0.0042 (16)
C9	0.051 (2)	0.037 (2)	0.049 (2)	−0.0035 (19)	0.0190 (18)	−0.0009 (16)
C10	0.048 (2)	0.040 (2)	0.047 (2)	−0.0014 (19)	0.0194 (17)	−0.0011 (16)
C11	0.050 (2)	0.039 (2)	0.048 (2)	−0.0033 (19)	0.0167 (17)	0.0026 (16)
C12	0.062 (2)	0.041 (2)	0.054 (3)	0.004 (2)	0.0163 (19)	0.0000 (17)
C13	0.094 (3)	0.043 (2)	0.076 (3)	−0.005 (2)	0.026 (2)	0.001 (2)

### Geometric parameters (Å, °)

**Table:**

N1—C1	1.487 (3)	C6—H6B	0.9700
N1—H1A	0.8900	C7—C8	1.523 (4)
N1—H1B	0.8900	C7—H7A	0.9700
N1—H1C	0.8900	C7—H7B	0.9700
O1—H1H	0.8499	C8—C9	1.515 (4)
O1—H1I	0.8499	C8—H8A	0.9700
C1—C2	1.513 (4)	C8—H8B	0.9700
C1—H1D	0.9700	C9—C10	1.520 (4)
C1—H1E	0.9700	C9—H9A	0.9700
C2—C3	1.518 (4)	C9—H9B	0.9700
C2—H2A	0.9700	C10—C11	1.517 (4)
C2—H2B	0.9700	C10—H10A	0.9700
C3—C4	1.523 (4)	C10—H10B	0.9700
C3—H3A	0.9700	C11—C12	1.506 (4)
C3—H3B	0.9700	C11—H11A	0.9700
C4—C5	1.524 (4)	C11—H11B	0.9700
C4—H4A	0.9700	C12—C13	1.522 (4)
C4—H4B	0.9700	C12—H12A	0.9700
C5—C6	1.522 (4)	C12—H12B	0.9700
C5—H5A	0.9700	C13—H13A	0.9600
C5—H5B	0.9700	C13—H13B	0.9600
C6—C7	1.515 (4)	C13—H13C	0.9600
C6—H6A	0.9700		
			
C1—N1—H1A	109.5	C6—C7—C8	114.8 (3)
C1—N1—H1B	109.5	C6—C7—H7A	108.6
H1A—N1—H1B	109.5	C8—C7—H7A	108.6
C1—N1—H1C	109.5	C6—C7—H7B	108.6
H1A—N1—H1C	109.5	C8—C7—H7B	108.6
H1B—N1—H1C	109.5	H7A—C7—H7B	107.5
H1H—O1—H1I	108.1	C9—C8—C7	114.9 (2)
N1—C1—C2	112.7 (3)	C9—C8—H8A	108.5
N1—C1—H1D	109.1	C7—C8—H8A	108.5
C2—C1—H1D	109.1	C9—C8—H8B	108.5
N1—C1—H1E	109.1	C7—C8—H8B	108.5
C2—C1—H1E	109.1	H8A—C8—H8B	107.5
H1D—C1—H1E	107.8	C8—C9—C10	115.0 (3)
C1—C2—C3	112.3 (3)	C8—C9—H9A	108.5
C1—C2—H2A	109.1	C10—C9—H9A	108.5
C3—C2—H2A	109.1	C8—C9—H9B	108.5
C1—C2—H2B	109.1	C10—C9—H9B	108.5
C3—C2—H2B	109.1	H9A—C9—H9B	107.5
H2A—C2—H2B	107.9	C11—C10—C9	115.1 (3)
C2—C3—C4	113.5 (3)	C11—C10—H10A	108.5
C2—C3—H3A	108.9	C9—C10—H10A	108.5
C4—C3—H3A	108.9	C11—C10—H10B	108.5
C2—C3—H3B	108.9	C9—C10—H10B	108.5
C4—C3—H3B	108.9	H10A—C10—H10B	107.5
H3A—C3—H3B	107.7	C12—C11—C10	115.1 (3)
C3—C4—C5	114.5 (3)	C12—C11—H11A	108.5
C3—C4—H4A	108.6	C10—C11—H11A	108.5
C5—C4—H4A	108.6	C12—C11—H11B	108.5
C3—C4—H4B	108.6	C10—C11—H11B	108.5
C5—C4—H4B	108.6	H11A—C11—H11B	107.5
H4A—C4—H4B	107.6	C11—C12—C13	115.0 (3)
C6—C5—C4	114.5 (3)	C11—C12—H12A	108.5
C6—C5—H5A	108.6	C13—C12—H12A	108.5
C4—C5—H5A	108.6	C11—C12—H12B	108.5
C6—C5—H5B	108.6	C13—C12—H12B	108.5
C4—C5—H5B	108.6	H12A—C12—H12B	107.5
H5A—C5—H5B	107.6	C12—C13—H13A	109.5
C7—C6—C5	114.8 (3)	C12—C13—H13B	109.5
C7—C6—H6A	108.6	H13A—C13—H13B	109.5
C5—C6—H6A	108.6	C12—C13—H13C	109.5
C7—C6—H6B	108.6	H13A—C13—H13C	109.5
C5—C6—H6B	108.6	H13B—C13—H13C	109.5
H6A—C6—H6B	107.6		
			
N1—C1—C2—C3	180.0 (3)	C6—C7—C8—C9	179.6 (3)
C1—C2—C3—C4	169.9 (3)	C7—C8—C9—C10	−179.8 (3)
C2—C3—C4—C5	−179.1 (3)	C8—C9—C10—C11	179.7 (3)
C3—C4—C5—C6	177.6 (3)	C9—C10—C11—C12	−179.7 (3)
C4—C5—C6—C7	−179.5 (3)	C10—C11—C12—C13	179.8 (3)
C5—C6—C7—C8	179.2 (3)		

### Hydrogen-bond geometry (Å, °)

**Table:**

*D*—H···*A*	*D*—H	H···*A*	*D*···*A*	*D*—H···*A*
N1—H1A···Cl1	0.89	2.44	3.303 (3)	162
N1—H1B···Cl1^i^	0.89	2.36	3.236 (2)	170
N1—H1C···O1^ii^	0.89	2.05	2.901 (4)	159
O1—H1H···Cl1	0.85	2.45	3.290 (3)	170
O1—H1I···Cl1^iii^	0.85	2.39	3.228 (2)	170

Symmetry codes: (i) *x*−1, −*y*+3/2, *z*−1/2; (ii) *x*, *y*, *z*−1; (iii) *x*−1, *y*, *z*.
